# Heteromeric TRPV4/TRPC1 channels mediate calcium-sensing receptor-induced relaxations and nitric oxide production in mesenteric arteries: comparative study using wild-type and TRPC1^−/-^ mice

**DOI:** 10.1080/19336950.2019.1673131

**Published:** 2019-10-11

**Authors:** Harry Z.E. Greenberg, Simonette R.E. Carlton-Carew, Alexander K. Zargaran, Kazi S. Jahan, Lutz Birnbaumer, Anthony P. Albert

**Affiliations:** a Vascular Biology Research Centre, Molecular & Clinical Sciences Research Institute, St. George’s, University of London, London, UK; b Laboratory of Neurobiology, National Institute of Environmental Health Sciences, Research Triangle Park, NC, USA; c Institute of Biomedical Research (BIOMED), Catholic University of Argentina, Buenos Aires, Argentina

**Keywords:** Calcium-sensing receptors, TRPC1, TRPV4, nitric oxide, endothelium, vascular smooth muscle

## Abstract

We have previously provided pharmacological evidence that stimulation of calcium-sensing receptors (CaSR) induces endothelium-dependent relaxations of rabbit mesenteric arteries through activation of heteromeric TRPV4/TRPC1 channels and nitric oxide (NO) production. The present study further investigates the role of heteromeric TRPV4/TRPC1 channels in these CaSR-induced vascular responses by comparing responses in mesenteric arteries from wild-type (WT) and TRPC1^-/-^ mice. In WT mice, stimulation of CaSR induced endothelium-dependent relaxations of pre-contracted tone and NO generation in endothelial cells (ECs), which were inhibited by the TRPV4 channel blocker RN1734 and the TRPC1 blocking antibody T1E3. In addition, TRPV4 and TRPC1 proteins were colocalised at, or close to, the plasma membrane of endothelial cells (ECs) from WT mice. In contrast, in TRPC1^-/-^ mice, CaSR-mediated vasorelaxations and NO generation were greatly reduced, unaffected by T1E3, but blocked by RN1734. In addition, the TRPV4 agonist GSK1016790A (GSK) induced endothelium-dependent vasorelaxations which were blocked by RN1734 and T1E3 in WT mice, but only by RN1734 in TRPC1^-/-^ mice. Moreover, GSK activated cation channel activity with a 6pS conductance in WT ECs but with a 52 pS conductance in TRPC1^-/-^ ECs. These results indicate that stimulation of CaSR activates heteromeric TRPV4/TRPC1 channels and NO production in ECs, which are responsible for endothelium-dependent vasorelaxations. This study also suggests that heteromeric TRPV4-TRPC1 channels may form the predominant TRPV4-containing channels in mouse mesenteric artery ECs. Together, our data further implicates CaSR-induced pathways and heteromeric TRPV4/TRPC1 channels in the regulation of vascular tone.

## Introduction

Stimulation of plasmalemmal calcium-sensing receptors (CaSR) by an increase in extracellular Ca^2+^ concentration ([Ca^2+^]_o_) is involved in maintaining plasma Ca^2+^ homeostasis through regulation of parathyroid hormone synthesis and secretion from the parathyroid gland, intestinal Ca^2+^ absorption, and renal Ca^2+^ excretion [1–3]. It is also apparent that CaSR are expressed in tissues not associated with plasma Ca^2+^ homeostasis, which suggests that CaSR are likely to be involved in regulating many different physiological systems.

In the vasculature, functional expression of CaSR in perivascular nerves, endothelial cells (ECs), and vascular smooth muscle cells (VSMCs) are proposed to regulate vascular tone and may be potential targets for controlling blood pressure [4–6]. However, there is currently little consensus on the overall function of CaSR in the vasculature, with studies suggesting that stimulation of CaSR induces both vasoconstriction and vasorelaxation through diverse cellular mechanisms [4–20]. In the presence of closely regulated plasma Ca^2+^ levels, stimulation of CaSR in the vasculature is considered possible as local [Ca^2+^]_o_ is likely to rise sufficiently at the surface of cells due to active Ca^2+^ transport mechanisms such as the Ca^2+^-ATPase and the Na^+^-Ca^2+^ exchanger [3,11,21].

We have previously shown that stimulation of CaSR by increasing [Ca^2+^]_o_ induces an endothelium-dependent relaxation in rabbit mesenteric arteries, which requires stimulation of the nitric oxide (NO)-guanylyl cyclase (GC)-protein kinase G (PKG) pathway coupled to activation of large conductance Ca^2+^-activated K^+^ (BK_Ca_) channels in VSMCs, and activation of intermediate conductance Ca^2+^-activated K^+^ (IK_Ca_) channels inducing endothelium-derived hyperpolarisations [16]. In a recent pharmacological study investigating how stimulation of CaSR may induce these mechanisms, we reported that CaSR-mediated activation of endothelial heteromeric TRPV4/TRPC1 channels is required for NO production whereas a different cation channel in ECs is likely to be involved in IK_Ca_ channel activation [20]. These data correspond with increasing evidence that TRPV4 channels have major roles in regulating vascular tone, including mediating flow- and agonist-induced vasodilatations via stimulation of NO production, and that TRPV4-mediated vascular responses are mediated by heteromeric TRPV4/TRPC1 channel structures expressed in ECs [23–27]. The present study provides further evidence for the role of heteromeric TRPV4-TRPC1 channels in CaSR-induced relaxations by comparing the effect of stimulating CaSR in mesenteric arteries from wild-type (WT) and TRPC1^-/-^ mice. Using wire myography, fluorescent microscopy, and electrophysiological techniques, our results suggest that heteromeric TRPV4/TRPC1 channels mediate CaSR-induced endothelium-dependent relaxations via NO production in mouse mesenteric arteries, and that TRPV4/TRPC1 channels may be the predominant TRPV4-containing channels present in mouse mesenteric artery ECs.

## Results

### CaSR-induced vasorelaxations and NO production are reduced in TRPC1^-/-^ mice

**Figure 1.**
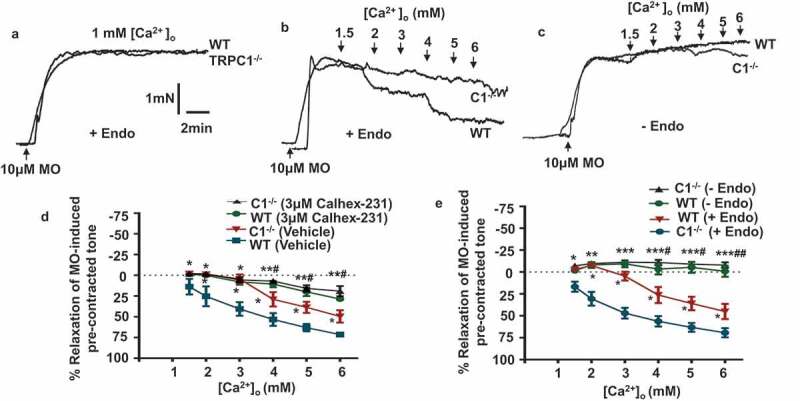
[Ca^2+^]_o_-induced relaxations of pre-contracted tone in mesenteric arteries from WT and TRPC1^-/-^ mice are mediated by CaSRs and endothelium. (a) Original traces showing that bath application of methoxamine (MO) induced sustained increases in tension in wild-type (WT) and TRPC1^-/-^ (C1^-/-^) vessel segments containing a functionally-intact endothelium. (b) Representative traces showing that increasing [Ca^2+^]_o_ from 1–6 mM induced relaxations of pre-contracted tone in both WT and C1^-/-^ vessels which contain a functional endothelium, but that responses in C1^-/-^ vessels were reduced. (c) Representative traces showing that [Ca^2+^]_o_-induced relaxations of pre-contracted tone were absent in both WT and C1^-/-^ vessels that did not contain a functionally-intact endothelium. (d and e) Mean data showing that [Ca^2+^]_o_-induced relaxations were smaller in C1^-/-^ compared to WT vessels, and that responses in both WT and C1^-/-^ vessels were reduced by Calhex-231 and absent following removal of a functional endothelium. Each point is from at least N = 6 animals, with at least n = 3 vessel segments from each animal. *Compares data to WT control, and ^#^compares data to C1^-/-^ control. One, two, and three symbols denote p < 0.05, p < 0.01, and p < 0.001 respectively.

We initially investigated if stimulating CaSR by increasing [Ca^2+^]_o_ produced similar endothelium-dependent relaxations of mouse mesenteric arteries to those described in rabbit mesenteric arteries [16]. In WT vessels, Figure 1(a–e) show that increasing [Ca^2+^]_o_ from 1–6 mM produced relaxations of pre-contracted tone induced by 10 μM methoxamine which were decreased by pre-treatment with the CaSR blocker Calhex-231, and were abolished by removal of a functional endothelium (Figure S2). Importantly, Figure 1(b–e) show that increasing [Ca^2+^]_o_ induced significantly smaller relaxations of TRPC1^-/-^ vessel segments compared to WT, although these relaxations were still inhibited by Calhex-231 and removal of a functional endothelium (Table 1). In control experiments, vasoconstrictions induced by 10 μM methoxamine and 60 mM KCl, and vasorelaxations induced by the NO donor SNP were similar in WT and TRPC1^-/-^ vessels (Figure 1(a) and S1), indicating that the general ability of vessels to contract and relax was not impaired in the absence of TRPC1.

**Figure 2.**
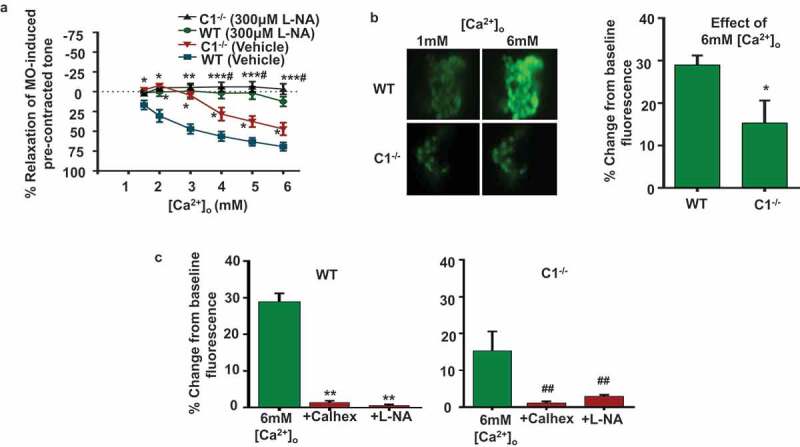
[Ca^2+^]_o_-induced relaxations of pre-contracted tone in mesenteric arteries from WT and TRPC1^-/-^ mice involve nitric oxide production in endothelial cells. (a) Mean data showing that [Ca^2+^]_o_-mediated relaxations of pre-contracted tone in WT and TRPC1^-/-^ vessels containing a functional endothelium were inhibited by pre-treatment with L-NAME (L-NA). Each point is from at least N = 6 animals, with at least n = 3 vessel segments from each animal. (b) Representative images and mean data showing that 6 mM [Ca^2+^]_o_ induced an increase in DAF-FM fluorescence in WT and TRPC1^-/-^ ECs, with a smaller response in TRPC1^-/-^ ECs. (c) Mean data showing that pre-treatment with Calhex-231 and L-NA inhibited increases in DAF-FM fluorescence from WT and TRPC1^-/-^ ECs induced by 6 mM [Ca^2+^]_o_. Each experiment from N = 6 animals, with n>50 cells per animal.

In our next experiments, we compared the role of NO in CaSR-induced vasorelaxations and NO production in WT and TRPC1^-/-^ preparations. Figure 2(a) shows that pre-treatment with the endothelial nitric oxide synthase inhibitor L-NAME (L-NA) inhibited CaSR-induced relaxations in WT and TRPC1^-/-^ vessels. In addition, Figure 2(b,c) reveal that increasing [Ca^2+^]_o_ from 1 mM to 6 mM increased baseline fluorescence of DAF-FM, a cell-permeable fluorescent NO indicator, by about 30% in WT ECs which was inhibited by pre-treatment with Calhex-231 and L-NA. In comparison, Figure 2(b,c) show that in TRPC1^-/-^ ECs, 6 mM [Ca^2+^]_o_ induced a smaller 15% increase in DAF-FM fluorescence, which was also inhibited by Calhex-231 and L-NA.

These results suggest that stimulation of CaSR induces endothelium-dependent relaxations involving NO production in mouse mesenteric arteries, and that these mechanisms involve TRPC1.

### TRPV4 and TRPC1 are both involved in CaSR-induced vasorelaxations and NO production

**Figure 3.**
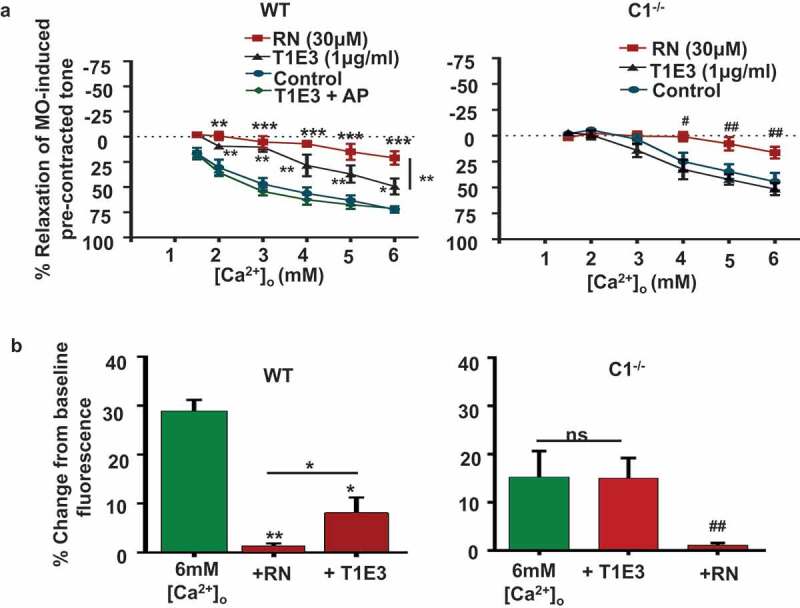
Effect of TRPV4 and TRPC1 channel blockers on [Ca^2+^]_o_-induced relaxations and nitric oxide production from WT and TRPC1^-/-^ mice. (a) Mean data showing that pre-treatment with RN1734 (RN) reduced [Ca^2+^]_o_-induced relaxations of pre-contracted tone in both WT and C1^-/-^ vessels, whereas T1E3 only attenuated [Ca^2+^]_o_-induced relaxations in C1^-/-^ vessels. Note that pre-incubation of T1E3 with its antigenic peptide (AgP) prevented the action of T1E3 on [Ca^2+^]_o_-induced relaxations in WT vessels. Each point from at least N = 6 animals, and at least n = 3 vessel segments per animal. (b) Mean data showing that RN reduced DAF-FM fluorescence induced by 6 mM [Ca^2+^]_o_ in WT and TRPC1^-/-^ ECs, whereas T1E3 only reduced responses in WT ECs. Each experiment from n = 6 animals, with n > 50 cells per animal.

To investigate the role of TRPV4 and TRPC1 channels in CaSR-induced vasorelaxations and NO production, we compared the effects of the TRPV4 blocker RN1734 (RN) and the TRPC1 blocking antibody T1E3 in WT and TRPC1^-/-^ mice. Figure 3(a,b) show that pre-treatment with RN produced a profound reduction in [Ca^2+^]_o_-induced relaxations of pre-contracted tone in both WT and TRPC1^-/-^ vessels, whereas pre-treatment with T1E3 only produced a reduction in [Ca^2+^]_o_-induced relaxations in WT vessels (Table 1). Pre-incubation of T1E3 with its antigenic peptide prevented the inhibitory effect of T1E3 on [Ca^2+^]_o_-induced relaxation in WT vessels. In addition, RN inhibited increases in baseline DAF-AM fluorescence induced by 6 mM [Ca^2+^]_o_ in both WT and TRPC1^-/-^ ECs, whereas T1E3 only reduced increases in [Ca^2+^]_o_-induced baseline fluorescence in WT ECs.

These results indicate that both TRPV4 and TRPC1 channels are required for CaSR-induced relaxations and NO production in mouse mesenteric arteries Interestingly, our findings suggest that CaSR-evoked TRPV4-containing channels can partially compensate in the absence of TRPC1.

### Heteromeric TRPV4-TRPC1 channels are expressed in mouse mesenteric artery ECs

**Figure 4.**
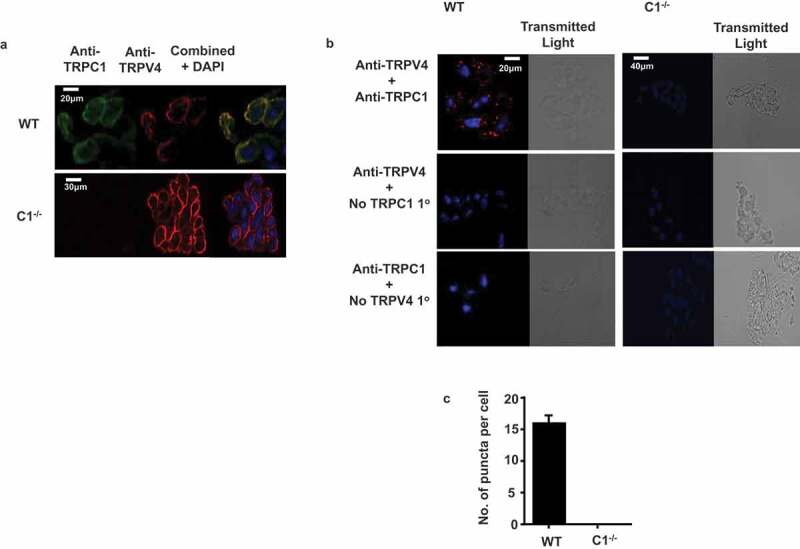
Expression and co-localization of heteromeric TRPV4-TRPC1 channels in mesenteric artery ECs. (a) Representative immunocytochemical images of TRPV4 (red) and TRPC1 (green) channel proteins in WT and TRPC1^-/-^ ECs, showing expression and co-localization (yellow) at, or close to, the plasma membrane. (b) Representative images and (c) mean data from PLA studies illustrating TRPV4 and TRPC1 co-localization puncta staining (red) in WT ECs, but not in TRPC1^-/-^ ECs. Data is from n = 4 animals, >10 cells per animal to calculate average number of puncta per cell.

We next examined the expression of TRPV4 and TRPC1, and potential heteromeric TRPV4/TRPC1 channel compositions in mesenteric artery ECs in WT and TRPC1^-/-^ mice. Figure 4(a) shows that TRPV4 and TRPC1 proteins were expressed in WT ECs using immunocytochemistry, with staining and pronounced co-localization present at, or close to, the plasma membrane. As expected, Figure 4(a) also reveals that TRPV4 was expressed at, or close to, the plasma membrane of TRPC1^-/-^ ECs but contained no detectable TRPC1 staining. Moreover, Figure 4(b,c) show that TRPV4 and TRPC1 form puncta signals using proximity ligation assay in WT but not TRPC1^-/-^ ECs indicating close spatial localization.

### TRPV4 and TRPC1 are both involved in GSK-induced vasorelaxations and cation channel activity

**Figure 5.**
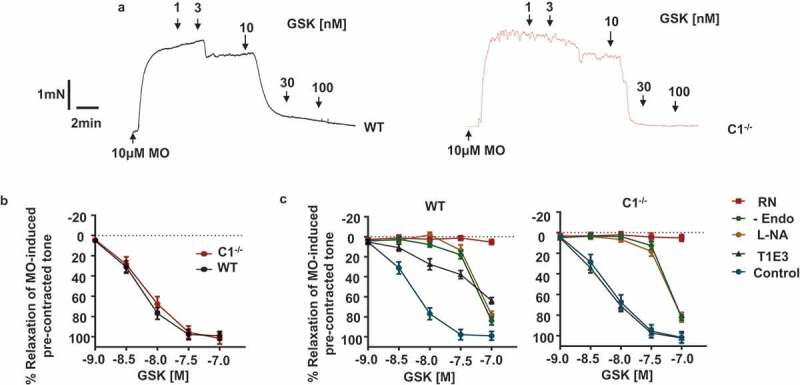
Effect of GSK1016790A on pre-contracted tone and NO production in WT and TRPC1^-/-^ mice. (a) Original traces and mean data showing that GSK1016790A (GSK)-induced relaxations of pre-contracted tone were similar in WT and C1^-/-^ vessels. WT EC_50_ was 4.6 ± 0.2nM vs. 4.9 ± 0.3nM in C1^-/-^ vessels whilst WT E_max_ was 98.9 ± 4.3% vs. 101.5 ± 5.4% in C1^-/-^ vessels. (b) Mean data showing that GSK-induced relaxations of pre-contracted tone were inhibited by RN1734 (RN) at all concentrations of GSK tested in WT and TRPC1^-/-^ vessels. In contrast, removal of a functional endothelium and pre-treatment with L-NA only inhibited responses induced by <100 nM GSK, and T1E3 only had an inhibitory action in WT compared to TRPC1^-/-^ vessels. Data from at least N = 6 animals, with at least n = 3 segments from each animal.

As the present study indicates that heteromeric TRPV4/TRPC1 channels mediate CaSR-induced vasorelaxations and NO production, we predicted that GSK101970A (GSK), a TRPV4 agonist, would induce vasorelaxations that are likely to be inhibited by TRPC1 blockade. Figure 5(a,b) illustrate that GSK induced pronounced relaxations of pre-contracted tone of WT and TRPC1^-/-^ mesenteric artery segments, which were reduced by removal of the endothelium, and by pre-treatment with either L-NAME or RN. Pre-treatment with T1E3 reduced GSK-induced vasorelaxations in WT vessels but had no effect on TRPC1^-/-^ vessels. Interestingly, GSK at high concentrations (e.g. 100 nM) was still able to induce full relaxations in WT and TRPC1^-/-^ vessels, which were maintained following removal of a functional endothelium (Figure 5 c).

**Figure 6.**
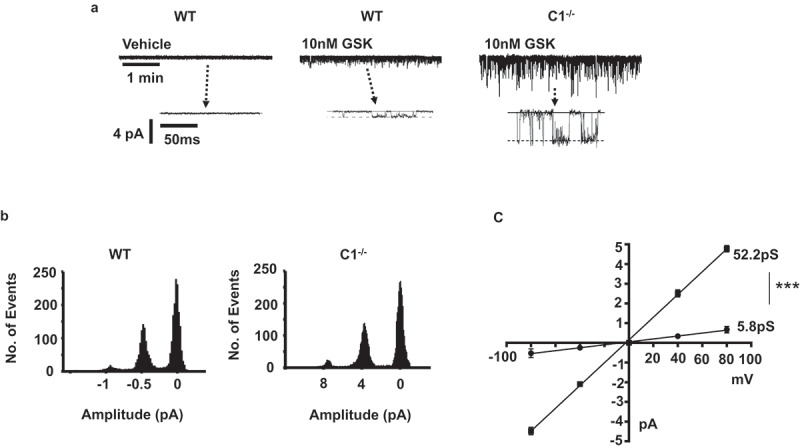
GSK1016790A-evoked cation channel currents in WT and TRPC1^-/-^ ECs. (a) Application of GSK1016790A (GSK) in the patch pipette solution activated cation channel activity in cell-attached patches held at −80 mV from WT and TRPC1^-/-^ ECs, with single channel current amplitudes being much greater in TRPC1^-/-^ ECs. (b) Representative amplitude histograms of GSK-induced single channel activity from WT and TRPC1^-/-^ ECs. Amplitude histograms had 3 identifiable peaks representing one closed (0 pA) and two unitary amplitude levels, namely ~-0.5 pA and −1pA in the WT patch vs. ~- 4pA and −8pA in the TRPC1^-/-^ EC patch. Given these values are multiples of each other it is likely that the respective patches contained at least 2 channels. (c) Mean pooled current/voltage relationships of GSK-evoked cation channel activities in WT and TRPC1^-/-^ ECs, which had unitary conductances of 5.8pS and 52.2pS respectively, and similar extrapolated reversal potentials of about 0 mV. Each data set is from at least n = 6 patches, from at least N = 5 animals, ***p < 0.001, 2-way ANOVA.

In our final experiments, we investigated the effect of GSK on cation channel activity at the single channel level in freshly isolated ECs. Figure 6(a,b) show that inclusion of 10 nM GSK in the patch pipette solution evoked cation channel activity in cell-attached patches from WT ECs, which had current amplitudes of about −0.5pA at −80 mV. This corresponded to a unitary conductance of about 6pS (Figure 6c). In contrast, Figure 6(a,b) reveal that GSK-induced cation channel activity in TRPC1^-/-^ ECs had current amplitudes of about −4pA at −80 mV and a unitary conductance of about 52pS (Figure 6c). Figure 6a also shows that cation channel activity was not recorded when GSK was absent from the patch pipette solution. In addition Figure 6(a,b) demonstrate that the GSK-evoked 52pS channel seen in TRPC1^-/-^ ECs was not observed in WT ECs.

These results provide further evidence that heteromeric TRPV4/TRPC1 contribute to GSK-induced vasorelaxations in mouse mesenteric arteries, and that these are the predominant TRPV4-containing channels in mouse mesenteric artery ECs. In addition, these data indicate that GSK activates other TRPV4-containing channels which are likely responsible for CaSR-mediated vasorelaxations and NO production observed in TRPC1^-/-^ preparations.

## Discussion

This comparative study using WT and TRPC1^-/-^ mice shows that stimulating CaSR induces endothelium-dependent relaxations via NO production in mesenteric arteries, which require activation of heteromeric TRPV4/TRPC1 channels in ECs. These findings complement our recent study that proposed a similar role for heteromeric TRPV4/TRPC1 channels in CaSR-induced vasorelaxations and NO production in rabbit mesenteric arteries[20]. In addition, the present study also provides further evidence that heteromeric TRPV4/TRPC1 channels may be the predominant TRPV4-containing channels in ECs[20].

### CaSR-induced relaxations and NO production in mouse mesenteric arteries are mediated by heteromeric TRPV4/TRPC1 channels

The present study shows that increasing [Ca^2+^]_o_ induces vasorelaxation of mesenteric arteries from WT mice, which are inhibited by the CaSR blocker Calhex-231. This confirms earlier studies indicating that stimulation of CaSR by increasing [Ca^2+^]_o_ induces relaxations in mouse mesenteric arteries [14,18], and also in rat and rabbit mesenteric arteries [10,16,17,20,28]. In addition, our results from WT mice illustrate that CaSR-induced vasorelaxations require an endothelium-dependent pathway involving endothelial nitric oxide synthase, and that stimulation of CaSR activates NO production in ECs. These findings confirm earlier studies in rabbit mesenteric arteries [16,20], but are different to studies using rat mesenteric arteries where an endothelium-independent role for perivascular nerves and cytochrome P450 metabolites is reported [7,8,10]. In conclusion, it seems clear that the predominant action of stimulating CaSR in mesenteric arteries is relaxation, although the pathways involved may be species-dependent.

The main aim of this study was to investigate an earlier proposal that heteromeric TRPV4/TRPC1 channels in ECs mediate CaSR-induced vasorelaxations and NO production in mesenteric arteries by comparing effects from WT and TRPC1^-/-^ mice. In WT mice, CaSR-induced vasorelaxations and NO generation in ECs were inhibited by RN and T1E3, and co-localization of TRPV4 and TRPC1 staining were present using immunocytochemical and PLA staining. In contrast, in TRPC1^-/-^ mice preparations, CaSR-induced vasorelaxations and NO production in ECs were reduced by about 50% compared to WT and not affected by T1E3, with the remaining responses inhibited by RN. These results collaborate with our previous findings in rabbit mesenteric arteries [16,20] to further indicate that endothelial CaSR-mediated pathways predominantly involve TRPV4/TRPC1-mediated Ca^2+^ influx and subsequent Ca^2+^-CaM-eNOS activity to induce the classical NO-GC-PKG pathway in the regulation of vascular tone.

The findings of the current study also support earlier studies indicating that heteromeric TRPV4/TRPC1 channels are expressed in ECs, which may also involve TRPP2 subunits [21, 23–27] making this channel unique by being composed of subunits from three different TRP subfamilies.

Several studies show that TRPC1-containing channels, including heteromeric TRPV4/TRPC1 channels in ECs, act as store-operated channels with TRPC1 likely to confer the ability of channels to be activated by store depletion via STIM1-mediated mechanisms in different cell types [21, 29–35]. Therefore, we might hypothesize that for optimal CaSR-induced vasorelaxations and NO generation to occur in WT mice and rabbit preparations, heteromeric TRPV4/TRPC1 channels are activated by store depletion-mediated pathways, but that different CaSR-mediated activation pathways of other TRPV4-containing channels are also present. This may explain how remaining TRPV4-containing channels mediating CaSR-induced vasorelaxations and NO production are able to partially compensate in TRPC1^-/-^ mice. In support of this hypothesis, a previous report showed that CCh-induced relaxation and NO production in aorta are reduced but not abolished in TRPC1^-/-^ mice[36]. In future studies, it will be therefore be important to determine if CaSR-induced heteromeric TRPV4/TRPC1 channel activation involves receptor stimulation via store depletion.

### GSK-evoked vasorelaxations are mediated by different TRPV4-containing channels in WT and TRPC1^-/-^ ECs

In rather surprising results, the concentration-response curves of GSK-induced relaxations were similar in mesenteric arteries from WT and TRPC1^-/-^ mice, and were inhibited to a similar extent by RN, L-NA, and removal of a functional endothelium whereas T1E3 only inhibited responses in WT mice. This suggests that GSK-activated heteromeric TRPV4/TRPC1 channels in WT and distinct TRPV4-containing channels in TRPC1^-/-^ mice via pathways that were equally potent at inducing vasorelaxations. Consistent with this conclusion, GSK activated a 6 pS cation channel in WT ECs and a 52 pS channel in TRPC1^-/-^ ECs which are likely to represent distinct TRPV4-containing channels. In our recent study in rabbit mesenteric artery ECs, GSK also activated a 6 pS cation channel which was inhibited by RN and T1E3 suggesting that this represented a channel composed of TRPV4 and TRPC1 subunits[20].

Notably, the current study did not reveal any obvious pharmacological differences between heteromeric TRPV4/TRPC1 channels in WT mice compared with TRPV4-containing channels in TRPC1^-/-^ mice. GSK was equally potent at inducing vasorelaxations of WT compared with TRPC1^-/-^ vessels and GSK-evoked NO-mediated responses were also equally sensitive to L-NA and to RN1734. Given that TRPV4 heteromisation with TRPC1 might be expected to determine distinct pharmacological features compared with TRPV4-containing channels in which TRPC1 is absent, in future studies it will be important to examine for potential pharmacological differences in greater detail e.g. by using different concentrations of RN1734 to inhibit channel activity, or by utilizing a range of other TRPV4 channel modulators not used in the current study – e.g. 4αPDD and HC067047.

Interestingly, high concentrations of GSK (e.g. 100 nM) were able to induce full vasorelaxations in WT and TRPC1^-/-^ mice in the presence of L-NA, T1E3, and removal of a functional endothelium. This suggests that either high concentration of GSK can overcome these inhibitory actions, e.g. there is enough functional endothelium remaining to induce relaxations, or that at high concentrations GSK may activate TRPV4-containing channels expressed on VSM to induce relaxations as had previously been reported[37].

Over-expression of TRPV4 and TRPC1 subunits and TRPV4-TRPC1 concatamers in HEK293 cells produced 4αPDD-evoked inward single channel activity with a unitary conductance of about 80pS[25], which is obviously very different to the 6pS channel conductance we recorded in WT ECs but similar to the 52pS channels in TRPC1^-/-^ ECs. These observations were surprising and the precise explanation for these differences remains unclear. It is possible that the electrophysiological properties of these channels are different in over-expression systems compared to native cells present in their physiological environment, or that the low 6pS conductance also reflects the presence of TRPP2 in the composition of the native channel. It will be important to investigate these differences in the future.

### Are heteromeric TRPV4-TRPC1 channels the predominant native TRPV4-containing channels in mesenteric artery ECs?

The present work only observed a 6pS channel composed on TRPV4 and TRPC1 subunits in WT ECs, which indicate that heteromeric TRPV4/TRPC1 channels are the predominant native TRPV4-containing channels in these cells. However, the TRPC1 blocker T1E3 was not as effective in reducing CaSR-induced vasorelaxations and NO production compared to RN1734 in preparations from WT mice, which may suggest that either other TRPV4-containing channels are present in WT ECs or that the T1E3 blocking antibody is less potent than a small molecular weight inhibitor. If other TRPV4-containing channels are present in WT ECs they are likely to be functionally expressed at low levels.

Sonkusare et al proposed that GSKapplication activated large amplitude Ca^2+^ sparklets, mediated by Ca^2+^ influx through a small number of rarely-opening TRPV4-containing channels that formed a cooperative cluster of about 4 channels. This in turn produces endothelium-dependent vasorelaxation via stimulation of SK_Ca_ and IK_Ca_ channels but not NO production in pressurized third-order mouse mesenteric arteries[38]. In contrast, the present work shows that GSK-induced vasorelaxation is mediated by NO generation in first-order mouse mesenteric arteries using wire myography. These disparities may represent differences between species, pressurized vessels and wire myography, vessel order, and activation of heteromeric TRPV4/TRPC1 6pS channels versus other TRPV4-containing channels, e.g. perhaps the 52pS channel. It would be interesting to investigate if the GSK-activated 6pS TRPV4/TRPC1 channels observed in rabbit mesenteric artery ECs could support enough Ca^2+^ entry to mediate Ca^2+^ sparklets, and if TRPC1 is involved in GSK-mediated Ca^2+^ sparklets and vasorelaxations in recorded in third-order mouse mesenteric artery ECs.

## Conclusion

This comparative study using WT and TRPC1^-/-^ mice provides further evidence that heteromeric TRPV4/TRPC1 channels are involved in CaSR-induced vasorelaxations through NO production in mesenteric arteries. These findings consolidate the proposals that vascular CaSR and heteromeric TRPV4/TRPC1 channels may represent novel therapeutic targets for regulating vascular tone and treating cardiovascular disease.

## Materials and methods

### Reagents

Goat anti-TRPV4 antibody (sc-47,527) characterized in several previous studies [27,39–41] was obtained from Santa Cruz Biotechnology (Dallas, Tx, USA) and T1E3, a rabbit anti-TRPC1 antibody, was generated by GenScript (Piscataway, NJ, USA) using a peptide sequence from a characterized putative extracellular pore region of the TRPC1 subunit[42]. Alexa Fluor 546-conjugated donkey anti-goat antibody (1:500) and Alexa Fluor 488-conjugated donkey anti-rabbit antibodies were obtained from Thermo Fisher Scientific (Walham, MA, USA). All drugs were purchased from Sigma-Aldrich (Sigma Chemical Co., Poole, UK) or Tocris (Tocris Biosciences, Bristol, UK). Drugs were dissolved in distilled water or dimethyl sulfoxide (DMSO).

### Animals

Wild-Type (WT) and TRPC1^-/−^ mice[43] aged 12–16 weeks and weighing 30–40 g, were used in this study. The initial mouse breeding colony was provided by the Comaprative Medicine Branch of the NIEHS (National Institute of Environmental Health Sciences, USA). The mice were housed in the Biological Research Facility (BRF) at St George’s University of London according to the requirements of the Code of Practice for animal husbandry, Animals Scientific Procedures Act 1986, amended in 2012. Animals were group-housed within appropriately sized single cages and room environmental conditions were controlled by an automated building management system that maintained a Light:Dark cycle of 12:12 hr, a room ambient temperature within a range of 18–22ºC, and a relative humidity of 50 ± 10%. They received ad lib fresh water and laboratory rodent diet (SDS, UK) and were provided with various environmental stimuli. All mice were killed by cervical dislocation, in accordance with Schedule I of the UK Animals Scientific Procedures Act, 1986 and St George’s University of London Animal Welfare and Ethical Review Committee.

### Cell and vessel segment preparation

First-order branches of the mouse superior mesenteric artery were micro-dissected and cleaned of adherent connective tissue and perivascular fat in physiological salt solution (PSS) containing (mM): NaCl 126, KCl 6, Glucose 10, HEPES 11, MgCl_2_ 1.2, and CaCl_2_ 1.5, pH adjusted to 7.2 with 10M NaOH. Mean resting vessel diameters in WT vs. TRPC1^-/−^ were similar (195 ± 6.3 vs. 197 ± 5.4 µm, n = 18 animals, >40 vessels per group)

Isolated vessels were then cut into 2 mm segments for wire myography experiments or first order and were dispersed to obtain freshly isolated ECs as previously described [16,20]. For EC isolation, vessels were washed in PSS containing 50 µM [Ca^2+^]_o_ for 5 min at 37°C and placed in collagenase solution (1 mg.ml^-1^) for 14 min at 37°C. Vessels were then triturated in fresh 50 µM [Ca^2+^]_o_ PSS and the cell-containing solution was collected and centrifuged for 1 min at 1000 rpm. The supernatant was removed and the cells re-suspended in fresh 0.75 mM [Ca^2+^]_o_ PSS, before plating onto coverslips. Isolated cells were then left at 4°C for 1 hr before use. Freshly isolated ECs were identified by their classic “cobblestone” morphology and “sheet-like” appearance[44].

### Immunocytochemistry

Freshly isolated mouse mesenteric artery ECs were fixed onto borosilicate coverslips with ice-cold 4% paraformaldehyde (Sigma-Aldrich, Gillingham, UK) for 10 min, washed 4 times with ice-cold phosphate-buffered saline (PBS), and permeabilised with PBS containing 0.1% Triton X-100 for 20 min at room temperature. Cells were then washed 4 times with ice-cold PBS and incubated with PBS containing 1% bovine serum albumin (BSA) for 1 hr at room temperature to block nonspecific antibody binding. The cells were then incubated overnight at 4°C with a TRPV4 antibody and T1E3. In control experiments, cells were incubated without primary antibodies. The following day, cells were washed 4 times with ice-cold PBS and incubated with secondary antibodies conjugated to fluorescent probes, Alexa Fluor 546-conjugated donkey anti-goat antibody or Alexa Fluor 488-conjugated donkey anti-rabbit antibodies (1:500). Unbound secondary antibodies were removed by washing with ice-cold PBS 4 times, and nuclei were labeled with 4ʹ,6-diamidino-2-phenylindole (DAPI) mounting medium (Sigma-Aldrich).

Cells were imaged using a Zeiss LSM 510 laser scanning confocal microscope (Carl Zeiss, Jena, Germany). Excitation was produced by stimulation with 543 nm or 488 nm lasers where appropriate, delivered to the specimen via a Zeiss Apochromat x63 oil-immersion objective. Emitted fluorescence was captured using LSM 510 software (release 3.2; Carl Zeiss). Two-dimensional images cut horizontally through the middle of the cells were captured and raw confocal imaging data processed using Zeiss LSM 510 software. Final images were produced using PowerPoint (Microsoft XP; Microsoft, Richmond, WA, USA).

### Proximity ligation assay

PLA[45] was performed using the Duolink in situ PLA detection kit 563 (Olink, Uppsala, Sweden) according to the manufacturer’s instructions. Freshly isolated mouse mesenteric artery ECs were fixed onto borosilicate coverslips with ice-cold 4% paraformaldehyde (Sigma-Aldrich, Gillingham, UK) for 10 min and then permeabilised in PBS containing 0.1% Triton X-100 for 20 min. ECs were blocked for 1 hr at 37°C using Duolink blocking solution to reduce nonspecific binding and were incubated overnight at 4°C with a TRPV4 antibody and T1E3 (both at dilution 1:200) in Duolink antibody diluent solution. Control experiments were carried out by omitting the primary antibodies. Secondary anti-goat PLUS and anti-rabbit MINUS antibody probes were then added for 1 hr at 37ºC. These specialized secondary antibodies with oligonucleotides attached, hybridize with additional oligonucleotides when in close proximity (<40 nm). Hybridized oligonucleotides were then ligated for 30 min at 37°C to form a closed DNA circle, before a rolling circle amplification step was initiated, lasting 100 min at 37°C. Complimentary oligonucleotides with a red fluorescence label attached were then hybridized to the products of the rolling circle amplification step and visualized as red puncta using a confocal LSM 510 microscope (Carl Zeiss). Mid-cell sections were selected, and the number of puncta were counted manually.

### Isometric tension recordings

Vessel segments 2 mm in length were mounted in a wire myograph (Model 610 M; Danish Myo Technology, Aarhus, Denmark) and equilibrated for 30 min at 37°C in 5 ml of gassed (95% O_2_/5% CO_2_) Krebs–Henseleit solution, composed of (mM): NaCl 118, KCl 4.7, MgSO_4_ 1.2, KH_2_PO_4_ 1.2, NaHCO_3_ 25, CaCl_2_ 2, D-glucose 10, pH 7.2. Vessels were normalized to 90% of the internal circumference predicted to occur under a transmural pressure of 100 mmHg[46]. After normalization, vessels were left for 10 min and were then challenged with 60 mM KCl for 5 min. Following a wash out with fresh Krebs solution, endothelium integrity was assessed by stably pre-contracting vessels with 10 µM methoxamine followed by the addition of 10 µM carbachol (CCh). Vessels in which CCh-induced vasorelaxations were >80% of pre-contracted tone were designated as having a functional endothelium intact. When required, endothelium was removed by rubbing the intima with a human hair. CCh-induced vasorelaxations of <10% of pre-contracted tone indicated successful removal. Vessel segments were then incubated for 30 min in fresh Krebs solution containing 1 mM CaCl_2_ followed by pre-contraction with 10 µM methoxamine. This was followed by cumulative additions of CaCl_2_ to increase [Ca^2+^]_o_ between 1–6 mM and thereby stimulate CaSR, or by cumulative additions of the selective TRPV4 agonist GSK1016790A (1–100 nM) in the presence of inhibitors tested or their respective vehicles. All inhibitors or their respective vehicles were added to the vessels 30 min before the construction of the concentration-response curves to [Ca^2+^]_o_ or GSK1016790A. For each experiment, vehicle controls were performed using vessel segments from the same animal.

### NO imaging

Experiments to visualize the production of NO in freshly isolated ECs were performed using the well-established cell-permeable fluorescent NO indicator DAF-FM diacetate [47,48]. Freshly dispersed ECs were placed in a sterilized 96-well plate and left for 1 hr at 4°C. Following this, ECs were loaded with 1 µM DAF-FM diacetate, incubated at 4°C for 20 min, and then washed with fresh PSS containing 1 mM [Ca^2+^]_o_. The cells were left for another 30 min at 4°C to allow complete de-esterification of intracellular diacetate. Inhibitors tested, or their respective vehicles were also added at this point. Changes in fluorescence following 5 min of CaSR stimulation with 6 mM [Ca^2+^]_o_ were captured using a Zeiss Axiovert 200M Inverted microscope. All experiments were performed at room temperature. The captured images were then processed and analyzed using AxioVision SE64 Software (Rel. 4.9.1; Carl Zeiss).

### Electrophysiology

Freshly isolated mouse mesenteric artery ECs from WT and TRPC1^-/-^ animals were used for recording single cation channel currents. Single channel activity was measured using the cell-attached configuration of the patch-clamp technique and recorded using an Axopatch 200B amplifier (Axon Instruments, Union City, CA, USA). Single cation channel currents were filtered at 100 Hz (−3 dB, low-pass 8-pole Bessel filter, Frequency Devices model LP02; Scensys, Aylesbury, UK) and sampled at 1 kHz (Digidata 1322A and pCLAMP 9.0 software; Molecular Devices, Sunnydale, CA, USA). All recordings were made in voltage-clamp mode at a holding potential of −40 mV and current/voltage (I/V) relationships were then evaluated by altering the holding potential between −80 and +80 mV.

The external bathing solution for cell-attached patch recordings contained in mM: 126 KCl, 1 CaCl_2_, 10 HEPES, and 11 glucose, adjusted to pH 7.2 with 10M KOH. The patch pipette solution contained (mM): 126 NaCl, 1 CaCl_2_, 10 HEPES, and 11 glucose adjusted to pH 7.2 with 10M NaOH. 100 μM DIDS, 100 μM niflumic acid, 10 mM TEA, 100 nM Apamin, and 100 nM CbTX were also included into block Ca^2+^ and swell-activated Cl^-^ conductances, voltage-gated K^+^ channels, and SK_Ca_, IK_Ca_, and BK_Ca_ channels respectively. This enabled cation conductances to be recorded in isolation. Single cation channel currents were activated by including 10 nM GSK1016790A in the patch pipette solution. All recordings took place at room temperature.

### Data and statistical analysis

Data points presented on all graphs and bar charts are mean values ± SEM, P < 0.05 was considered a significant difference between groups. For wire myography experiments, all relaxant responses are expressed as percentage relaxation of stable precontracted tone induced by 10 µM methoxamine. Responses to increasing concentrations of [Ca^2+^]_o_ and GSK101790A in treated vs. control vessels for both wild-type and TRPC1^-/-^ mice were analyzed by 2-way ANOVA followed by Bonferroni *post hoc* tests. Bonferroni comparisons are shown above the graph data points whereby: * P < 0.05, ** P < 0.01, *** P < 0.001, **** P < 0.0001 vs. control. Statistical analysis and graphs were made using Graphpad Prism 6 software (GraphPad Software, Inc, San Diego, CA, USA).

For NO imaging experiments, changes in DAF-FM fluorescence before and after CaSR stimulation were quantified by selecting a cell as a region of interest (ROI). Changes in fluorescence levels within the ROI before and after the experimental protocols were analyzed using unpaired Student’s *t*-test. Figures and analysis were made using Graphpad Prism 6 (GraphPad Software, Inc, San Diego, CA, USA).

For analysis of single-channel recordings, current amplitudes were calculated from traces of ≥ 60 s in duration using the 50% threshold method. Amplitude histograms were fitted with gaussian curves using a bin width of 0.01pA and 0.05pA for WT and TRPC1-/- patches respectively, with peak values corresponding to channel open levels. Mean channel amplitudes at respective membrane potentials between −80 and +80 mV were plotted, enabling current/voltage (I/V) relationships to be fitted by linear regression, with the gradient determining conductance values. Figures and analyses were prepared using MicroCal Origin 6.0 software (MicroCal Software, Northampton, MA, USA), in which inward single-channel openings are shown as downward deflections. *P < 0.05, **P < 0.01, ***P < 0.001, ****P < 0.0001 vs. relevant controls.
