# Supplementary material for: Heteromeric TRPV4/TRPC1 channels mediate calcium-sensing receptor-induced relaxations and nitric oxide production in mesenteric arteries: comparative study using wild-type and TRPC1−/- mice
Source: Channels (Austin). 2019 Oct 11;13(1):410–23. doi: 10.1080/19336950.2019.1673131 (PMC7426016; doi:10.1080/19336950.2019.1673131)
Supplement: Supplemental Material [file kchl-13-01-1673131-s001.pptx]

## Slide 1
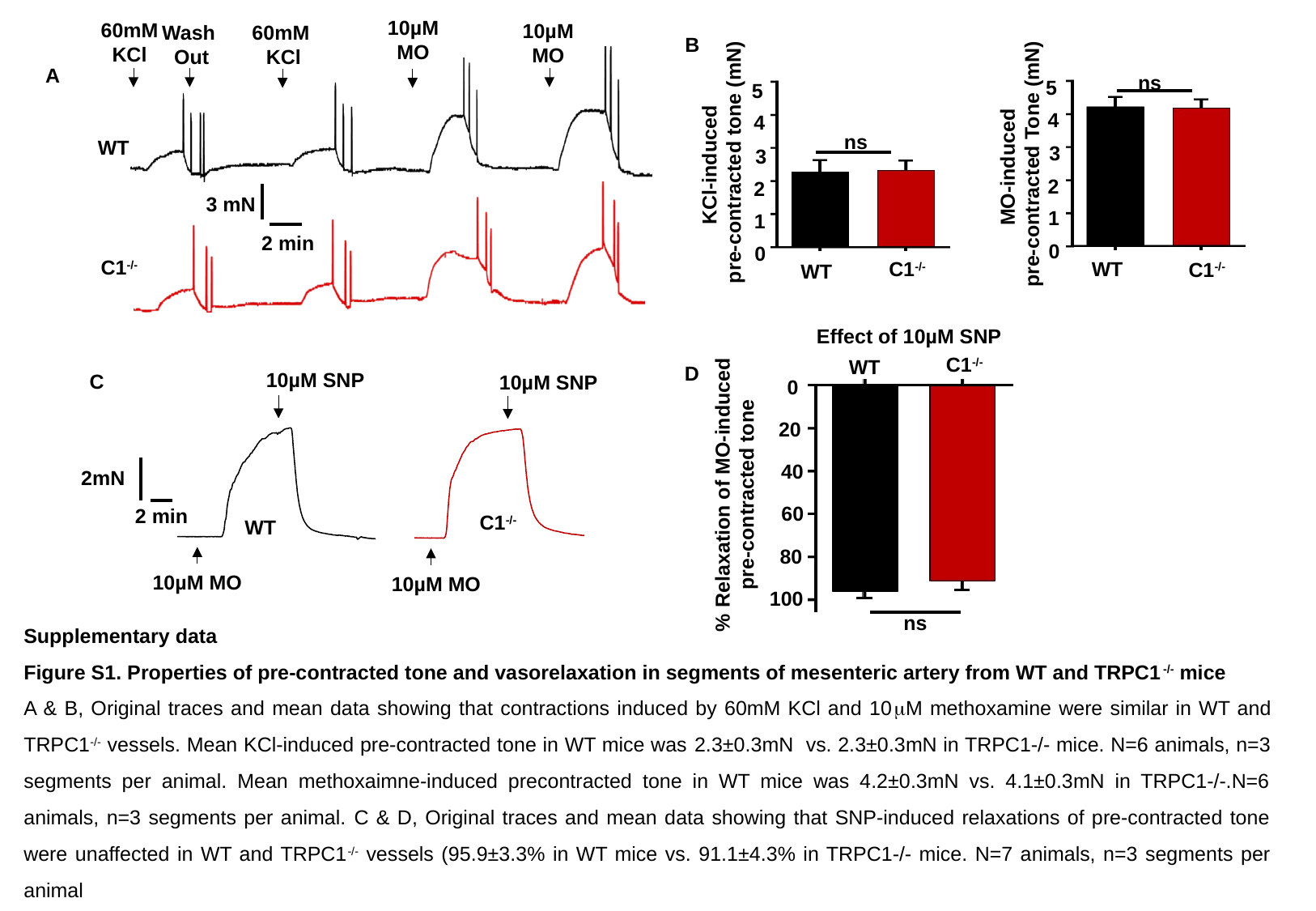

10µM
MO
60mM
KCl
10µM
MO
Wash
Out
60mM
KCl
A
WT
3 mN
2 min
C1-/-
B
ns
5
5
 4
 4
ns
 3
 3
KCl-induced
 pre-contracted tone (mN)
MO-induced
pre-contracted Tone (mN)
 2
 2
 1
 1
 0
 0
WT
C1-/-
C1-/-
WT
Effect of 10µM SNP
C1-/-
WT
D
10µM SNP
10µM SNP
10µM MO
2mN
2 min
C1-/-
WT
10µM MO
C
0
 20
40
% Relaxation of MO-induced
pre-contracted tone
 60
 80
 100
ns
Supplementary data
Figure S1. Properties of pre-contracted tone and vasorelaxation in segments of mesenteric artery from WT and TRPC1-/- mice
A & B, Original traces and mean data showing that contractions induced by 60mM KCl and 10μM methoxamine were similar in WT and TRPC1-/- vessels. Mean KCl-induced pre-contracted tone in WT mice was 2.3±0.3mN vs. 2.3±0.3mN in TRPC1-/- mice. N=6 animals, n=3 segments per animal. Mean methoxaimne-induced precontracted tone in WT mice was 4.2±0.3mN vs. 4.1±0.3mN in TRPC1-/-.N=6 animals, n=3 segments per animal. C & D, Original traces and mean data showing that SNP-induced relaxations of pre-contracted tone were unaffected in WT and TRPC1-/- vessels (95.9±3.3% in WT mice vs. 91.1±4.3% in TRPC1-/- mice. N=7 animals, n=3 segments per animal

## Slide 2
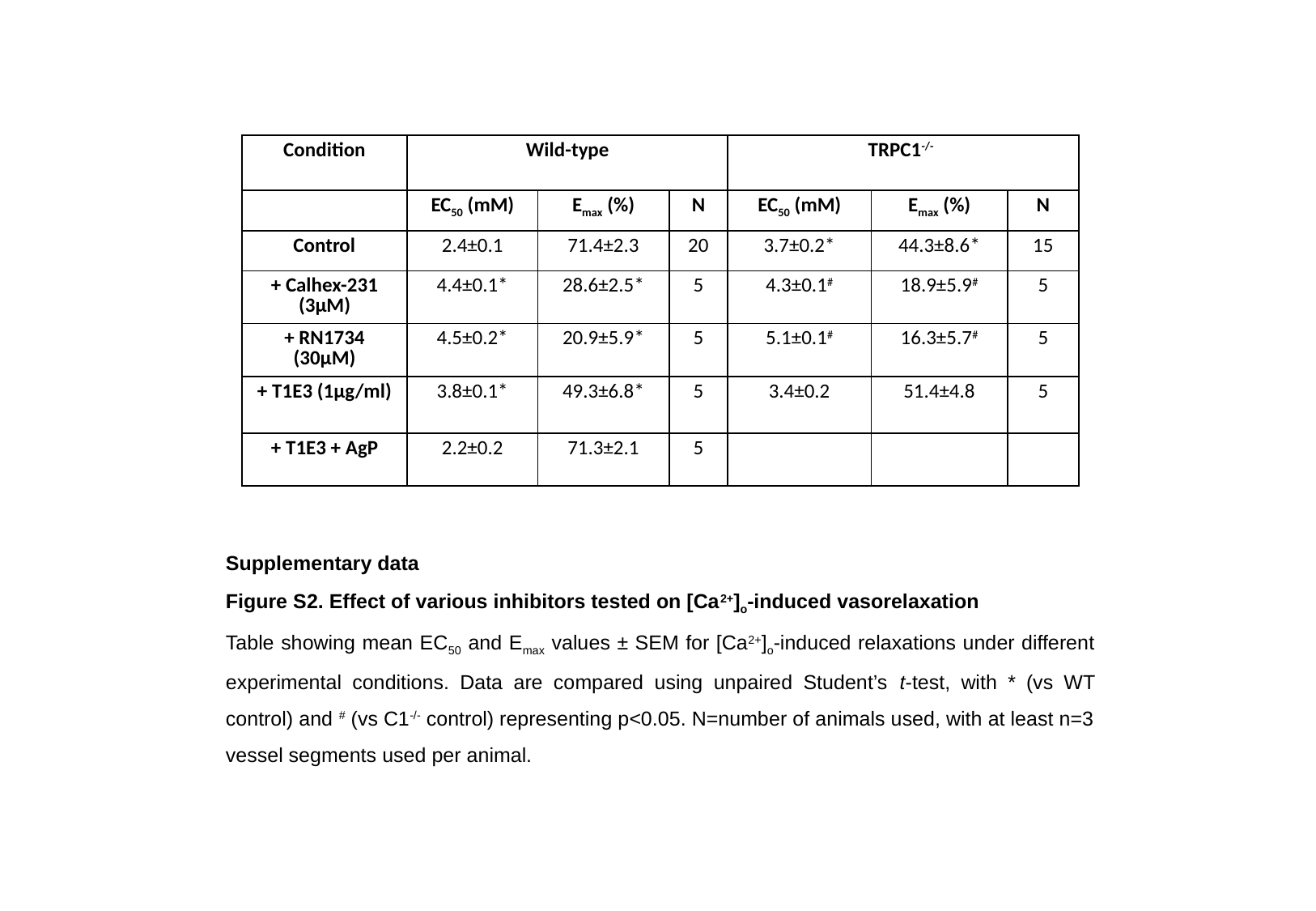

| Condition | Wild-type | | | TRPC1-/- | | |
| --- | --- | --- | --- | --- | --- | --- |
| | EC50 (mM) | Emax (%) | N | EC50 (mM) | Emax (%) | N |
| Control | 2.4±0.1 | 71.4±2.3 | 20 | 3.7±0.2\* | 44.3±8.6\* | 15 |
| + Calhex-231 (3µM) | 4.4±0.1\* | 28.6±2.5\* | 5 | 4.3±0.1# | 18.9±5.9# | 5 |
| + RN1734 (30µM) | 4.5±0.2\* | 20.9±5.9\* | 5 | 5.1±0.1# | 16.3±5.7# | 5 |
| + T1E3 (1µg/ml) | 3.8±0.1\* | 49.3±6.8\* | 5 | 3.4±0.2 | 51.4±4.8 | 5 |
| + T1E3 + AgP | 2.2±0.2 | 71.3±2.1 | 5 | | | |
Supplementary data
Figure S2. Effect of various inhibitors tested on [Ca2+]o-induced vasorelaxation
Table showing mean EC50 and Emax values ± SEM for [Ca2+]o-induced relaxations under different experimental conditions. Data are compared using unpaired Student’s t-test, with * (vs WT control) and # (vs C1-/- control) representing p<0.05. N=number of animals used, with at least n=3 vessel segments used per animal.
